# Treatment patterns in patients with castration-resistant prostate cancer who received darolutamide in the ARAMIS trial in Spain: PARASEC study

**DOI:** 10.1007/s12094-026-04233-8

**Published:** 2026-02-10

**Authors:** Javier Puente, Rubén Campanario, David Marmolejo, Juan Andrés Cantero-Mellado, Álvaro Gómez-Ferrer, Alfredo Rodríguez Antolín, María J. Ribal, Natalia Picola Brau, María José Ledo, Carlos Hernandez, Carlos Llorente, Carmen González-Enguita, Álvaro Bisonó Castillo, Joan Benejam, Jesús Gil Guijarro, Jose Garcia-Sanchez, Joan Folqué, Javier Casas-Nebra

**Affiliations:** 1 Department of Medical Oncology, Hospital Clínico San Carlos, C/Profesor Martin Lagos S/N, 28040 Madrid, Spain; 2 Department of Urology, Hospital Universitario Virgen del Rocío, Sevilla, Spain; 3 Department of Oncology, Hospital Universitari Vall d’Hebron, Barcelona, Spain; 4 Department of Urology, Hospital Clínico Universitario Virgen de La Victoria, Málaga, Spain; 5 Department of Urology, Instituto Valenciano de Oncología, Valencia, Spain; 6 Department of Urology, Hospital Universitario, 12 de Octubre, Madrid, Spain; 7 Uro-Oncology Unit, Hospital Clínic de Barcelona, Barcelona, Spain; 8 Department of Urology, Bellvitge University Hospital, Barcelona, Spain; 9 Department of Urology, Hospital Universitario Puerta del Mar, Cádiz, Spain; 10 Department of Urology, Gregorio Marañón University General Hospital, Madrid, Spain; 11 Department of Urology, Hospital Universitario Fundacion Alcorcon, Alcorcón, Spain; 12 Department of Urology, Hospital Universitario Fundación Jiménez Díaz, UAM, Madrid, Spain; 13 Hospital Universitario de Jerez de la Frontera, Jerez De La Frontera, Spain; 14 Department of Urology, Hospital de Manacor, Mallorca, Spain; 15 Department of Urology, Hospital General Universitario de Elche, Elche, Spain; 16 Department of Oncology, Hospital Arnau de Vilanova, Valencia, Spain; 17 Bayer Hispania, S.L., Sant Joan Despí (Barcelona), Spain; 18 Department of Urology, Hospital Universitario Lucus Augusti, Lugo, Spain

**Keywords:** nmCRPC, mCRPC, Real-world evidence, Darolutamide, Abiraterone, Docetaxel, Cabazitaxel

## Abstract

**Purpose:**

We aimed to describe treatment patterns of patients with nonmetastatic castration-resistant prostate cancer (nmCRPC) who progressed after receiving darolutamide in a real-world setting, and according to the standard clinical practice in Spain.

**Methods:**

This was a multicenter, observational, retrospective study conducted at the urology and oncology departments of 17 Spanish hospitals that participated in the ARAMIS trial and its rollover study.

**Results:**

85 patients, with a median age of 76 years, were included in the study. 49 patients (57.6%) progressed to mCRPC, with metastases located mainly in bone. Only 35 of them (71.4%) received at least one subsequent therapy. The most common first-line treatments after darolutamide were abiraterone (*n* = 22, 63%) and docetaxel (*n* = 10, 29%), with a median (IQR) treatment duration of 7.6 months (4.7, 12.7) and 4.8 months (3.8, 5.8), respectively; besides, the most frequent first-line/second-line treatment sequences were abiraterone–docetaxel and docetaxel–cabazitaxel. In addition, only 20% of patients with bone metastases received osteoclast-targeted therapy.

**Conclusion:**

These real-world practice patterns suggest a lack of consensus in Spanish clinical practice for the management of patients with mCRPC, indicating that there is a need for more standardized strategies and unification of the criteria to make decisions in accordance with the recommendations of international clinical practice guidelines.

**Clinical trial registration:**

Not applicable.

**Supplementary Information:**

The online version contains supplementary material available at 10.1007/s12094-026-04233-8.

## Introduction

Second-generation androgen receptor pathway inhibitors (ARPIs), such as apalutamide, enzalutamide, and darolutamide, are considered the standard of care for patients with high-risk nonmetastatic castration-resistant prostate cancer (nmCRPC) [1–4]. Despite their availability, most patients with nmCRPC eventually progress to metastatic disease [4, 5]. The treatment landscape for nmCRPC is rapidly evolving, with many effective treatment options for mCRPC that have been progressively approved and adopted by guidelines [2, 3, 6]. Among these, second-generation antiandrogens (abiraterone and enzalutamide), taxane-based chemotherapy (docetaxel and cabazitaxel), poly ADP‒ribose polymerase (PARP) inhibitors (olaparib, niraparib, and talazoparib), radiopharmaceutical agents (radium-223 and 177Lutetium-PSMA-617), and immunotherapy (sipuleucel-T) have been shown to improve OS in patients with mCRPC [7–10]. However, the best options for first-line therapy for mCRPC and the sequence of further lines in this setting are unclear as optimal treatment selection is complex and depends on several factors, such as disease burden, patient comorbidities, tumor characteristics, sites of metastases, history of prior treatments, drug side effects, the presence of genomic alterations, and accessibility to treatment in the entire health system [8, 9, 11, 12].

Importantly, there is a lack of data regarding the treatment patterns of patients with nmCRPC who progress after receiving darolutamide in a real-world setting. This retrospective study aims to describe the treatments received by patients included in Spanish sites that participated in the ARAMIS study, the pivotal study supporting the efficacy of darolutamide in patients with nmCRPC [13, 14]. Specifically, it focuses on treatments administered after treatment with darolutamide according to standard clinical practice in Spain. In addition, the present study evaluates whether those treatment patterns are concordant with guidelines.

## Patients and methods

### Study design

This was a multicenter, observational, noninterventional, retrospective, descriptive, follow-up study conducted at the Urology and Oncology Departments of 17 selected Spanish hospitals that participated in the ARAMIS and its rollover trial. ARAMIS was an international, double-blind, placebo-controlled trial conducted in 1509 men with nmCRPC receiving 600 mg of darolutamide twice daily in combination with andrigen-deprivation therapy (ADT) versus placebo plus ADT. Upon completion of the double-blind treatment period, 41% of the patients in the darolutamide arm continued with the drug in an open-label fashion. Similarly, 31% of the patients in the placebo group crossed over to receive open-label darolutamide (ROS study) [14, 15]. Spanish investigators contributed 8% of the overall ARAMIS study population, with 75 subjects randomized to the darolutamide group and 42 to the placebo group. In addition, 16 patients from the placebo arm crossed over to receive open-label darolutamide (rollover study [ROS]) after the unblinding of the treatment assignments, resulting in a total of 91 patients treated with darolutamide [13].

The data collection for this study took place between November 2023 and March 2024, and the patient follow-up covered a span of 10 years, extending from 2014 (first patient’s first visit) to 2023 (last patient’s last visit) (Supplementary Fig. 1).

This study was reviewed and approved by the Regional Independent Reference Ethics Committee of Galicia, Spain (CEIC 2023/371). This study followed the Guidelines for Good Pharmacoepidemiology Practices of the International Society for Pharmacoepidemiology [16] and Good Clinical Practice standards [17]. Informed consent was obtained from the enrolled patients whenever feasible (i.e., when the patient died or was lost to follow-up, it was not mandatory to obtain informed consent), according to the applicable Spanish regulations (Royal Decree 957/2020) [18].

### Study population

Patients with nonmetastatic castration-resistant prostate cancer (nmCRPC) who participated in the ARAMIS study at Spanish sites and who received darolutamide in either the ARAMIS or ROS studies were included. Patients who died or were lost to follow-up after starting darolutamide treatment were included.

The inclusion criteria for patients enrolled in ARAMIS were men ≥ 18 years of age, diagnosed with nmCRPC by conventional imaging (bone scan and CT), with a baseline prostate-specific antigen (PSA) level ≥ 2 ng/mL, a PSA doubling time (PSADT) ≤ 10 months, and an Eastern Cooperative Oncology Group (ECOG) performance status (PS) of 0–1 [19].

### Study objectives, data sources, and assessments

The primary objective of this study was to describe the patterns of treatment after darolutamide treatment in nmCRPC patients who progressed to mCRPC, with a focus on subsequent treatments and sequences.

The secondary study objectives were as follows: (1) to describe the proportion of patients with nmCRPC who received darolutamide in the ARAMIS study and progressed to mCRPC until the last anticancer treatment; (2) to describe the sequence of administration of mCRPC treatments after darolutamide according to standard clinical practice in Spain; (3) to describe the duration of treatment after darolutamide and reasons for treatment discontinuation; (4) to describe the number of mCRPC treatment lines received after darolutamide; and (5) to describe whether mCRPC patients received palliative radiotherapy and/or osteoclast-targeted therapy (denosumab or bisphosphonates) after darolutamide.

The source of the study information was the dataset of the ARAMIS and ROS studies for baseline patient demographic and clinical characteristics and the available existing data in the patient medical records at the different participating sites for the follow-up data.

The following baseline variables were included in the study assessment: age, Eastern Cooperative Oncology Group (ECOG) Performance Status, disease characteristics at ARAMIS study inclusion (including PSA level), PSA doubling time (PSADT), presence of regional pathologic lymph nodes, nmCRPC diagnosis date, disease characteristics at PC diagnosis (including PC diagnosis date), Gleason score, clinical and pathological cancer stage, PSA level, and previous therapies for PC (e.g., hormonal treatment, radiotherapy, and surgery).

For the primary objective, the anticancer treatments received by the study patients after the completion of the ARAMIS and ROS studies were recorded. In addition, for the secondary endpoints, the following variables were also recorded: date of progression to mCRPC, location of metastasis at progression of the PC after the end of the ARAMIS and ROS studies (distant lymph nodes, bone, visceral, and other sites), palliative radiotherapy utilization with the start/end dates, osteoclast-targeted therapy administration (bisphosphonate or denosumab) and start/end dates, darolutamide treatment after the end of the ARAMIS and ROS studies (treatment duration, dose reductions, re-escalations, and interruptions), and the total number, type, and line sequences of the treatments for the metastatic disease.

### Statistical analysis

No formal sample size calculations were performed. The original eligible study population consisted of 91 available Spanish patients with nmCRPC who participated in the ARAMIS/ROS study and were treated with darolutamide.

Given the nature and objectives of this study, the statistical methodology applied was mainly descriptive. For continuous variables, the means and standard deviations (SDs) and/or medians and interquartile ranges (IQRs) are reported. Categorical variables are presented as absolute (*n*) and relative frequencies (%).

The start date of darolutamide therapy during the ARAMIS trial or ROS study was considered the index date. Metastasis-free survival (MFS), defined as the time from the index date to the date of metastasis or death, whichever occurred first, was estimated using the Kaplan–Meier (KM) method, including the median survival time and its 95% confidence interval. Patients who did not progress to mCRPC were censored on the last available follow-up date.

Statistical analysis was performed using IBM SPSS Statistics version 26 (IBM Corp., Armonk, NY, USA).

## Results

Eighty-five patients with nmCRPC from 17 Spanish sites who participated in the ARAMIS and ROS studies were included in the PARASEC study (Supplementary Fig. 2 and Supplementary Table 1). At the time of darolutamide initiation, the median (IQR) age was 76 years (72.0, 82.0), and 85% of the patients had an Eastern Cooperative Oncology Group (ECOG) performance status of 0. In addition, the patients had a median (IQR) PSA of 12.5 ng/mL (6.8, 21.1) and a median (IQR) PSADT of 5.1 months (3.6, 6.5), and only 3.5% of them presented regional lymph nodes. After ARAMIS study completion, open-label darolutamide was administered to 85 patients included in the PARASEC study who received this treatment for a total median (IQR) time of 38.9 months (14.6, 62.0). The darolutamide dose was reduced in 10 patients (11.8%), interrupted in 25 patients (29.4%), and re-escalated in 24 patients (28.2%). The baseline demographic and clinical patient characteristics are shown in Table 1.

**Table 1:** Baseline demographic and clinical characteristics

Characteristic	*N*	
Age, years, median (IQR)	85	76.0 (72.0, 82.0)
Racial group, *n* (%)	85	
Caucasian		84 (98.8)
Hispanic		1 (1.2)
ECOG-PS at darolutamide initiation, *n* (%)	85	
0		72 (84.7)
1		13 (15.3)
Regional lymph nodes at darolutamide initiation, *n* (%)	85	3 (3.5)
PSA at ARAMIS inclusion, ng/mL, median (IQR)	85	12.5 (6.8, 21.1)
PSADT at ARAMIS inclusion, months, median (IQR)		5.1 (3.6, 6.5)
< 6 months, *n* (%)	85	58 (68.2)
TNM stage at ARAMIS inclusion, *n* (%)	58	
I		5 (8.6)
IIA		7 (12.1)
IIB		3 (5.2)
IIC		9 (15.5)
IIIA		11 (19.0)
IIIB		10 (17.2)
IVA		1 (1.7)
NA		12 (2.7)
PSA at PC diagnosis, ng/mL, median (IQR)	75	19.0 (9.1, 52.4)
Gleason score ≥ 7 at PC diagnosis, *n* (%)	81	64 (79.0)
Prior hormone therapy to ARAMIS inclusion, *n* (%)	85	85 (100%)
Prior radiotherapy to ARAMIS inclusion, *n* (%)	85	46 (54.1%)
Prior surgery to ARAMIS inclusion, *n* (%)	85	23 (27.1)

*ECOG-PS* European Cooperative Oncology Group performance status, *IQR* interquartile range, *N* number of evaluable patients, *PC* prostate cancer, *PSA* prostate-specific antigen, *PSADT* prostate-specific antigen doubling time, *TNM* TNM classification of malignant tumors

### Subsequent treatments in patients who progressed to mCRPC after treatment with darolutamide

Forty-nine patients (57.6%) in the study population progressed to mCRPC, 35 (71.4%) of whom underwent treatment for metastatic disease. The median (IQR) number of treatment lines administered for mCRPC was 2 (1.0, 5.0) (Table 2). The drugs most frequently used for mCRPC in the first-line setting (*n* = 35) were abiraterone in 22 patients (63%), docetaxel in 10 patients (29%), and enzalutamide in 2 patients (5.7%). In the second-line setting (*n* = 21), the most common treatments were docetaxel in 12 patients (57%) and cabazitaxel in 4 patients (19%). Finally, in the third-line setting (*n* = 12), the most common treatment was cabazitaxel in 4 patients (33%) and docetaxel in 3 patients (25%) (Table 2). Throughout the entire follow-up period, radium-223 was administered to five patients (14.3%), niraparib was administered to two patients (5.7%), one patient received radium-223 in combination with abiraterone in the first-line, and one patient received radium-223 in combination with an anti-PD-1 agent in the third-line (Table 2).

**Table 2:** Treatment scheme for patients with mCRPC after treatment with darolutamide

Line number	Scheme line	*n*	%
1	Abiraterone	20	57.1
	Docetaxel	8	22.9
	Enzalutamide	2	5.7
	Docetaxel + abiraterone	1	2.9
	Docetaxel + cabazitaxel	1	2.9
	Carboplatin + pemetrexed	1	2.9
	Carboplatin + etoposide + cyclophosphamide	1	2.9
	Abiraterone + niraparib	1	2.9
	Total	35	100.0
2	Docetaxel	12	57.1
	Cabazitaxel	4	19.0
	Radium-223	2	9.5
	Abiraterone	1	4.8
	Cyclophosphamide	1	4.8
	Atezolizumab + cabozantinib	1	4.8
	Total	21	100.0
3	Cabazitaxel	4	33.3
	Docetaxel	3	25.0
	Radium-223	2	16.7
	Cyclophosphamide	1	8.3
	Niraparib + AntiPD1	1	8.3
	Enzalutamide + prednisone	1	8.3
	Total	12	100.0
4	Cabazitaxel	3	42.9
	Cyclophosphamide	2	28.6
	Radium-223	1	14.3
	Carboplatin	1	14.3
	Total	7	100.0
5	Enzalutamide	2	66.7
	Cabazitaxel	1	33.3
	Total	3	100.0

*n* absolute frequency, *PD1* programmed cell death protein 1

Treatment schemes are described with a “+” when active substances were combined in the same scheme and with a “/” when there was a switch to a different scheme

**Fig. 1:**
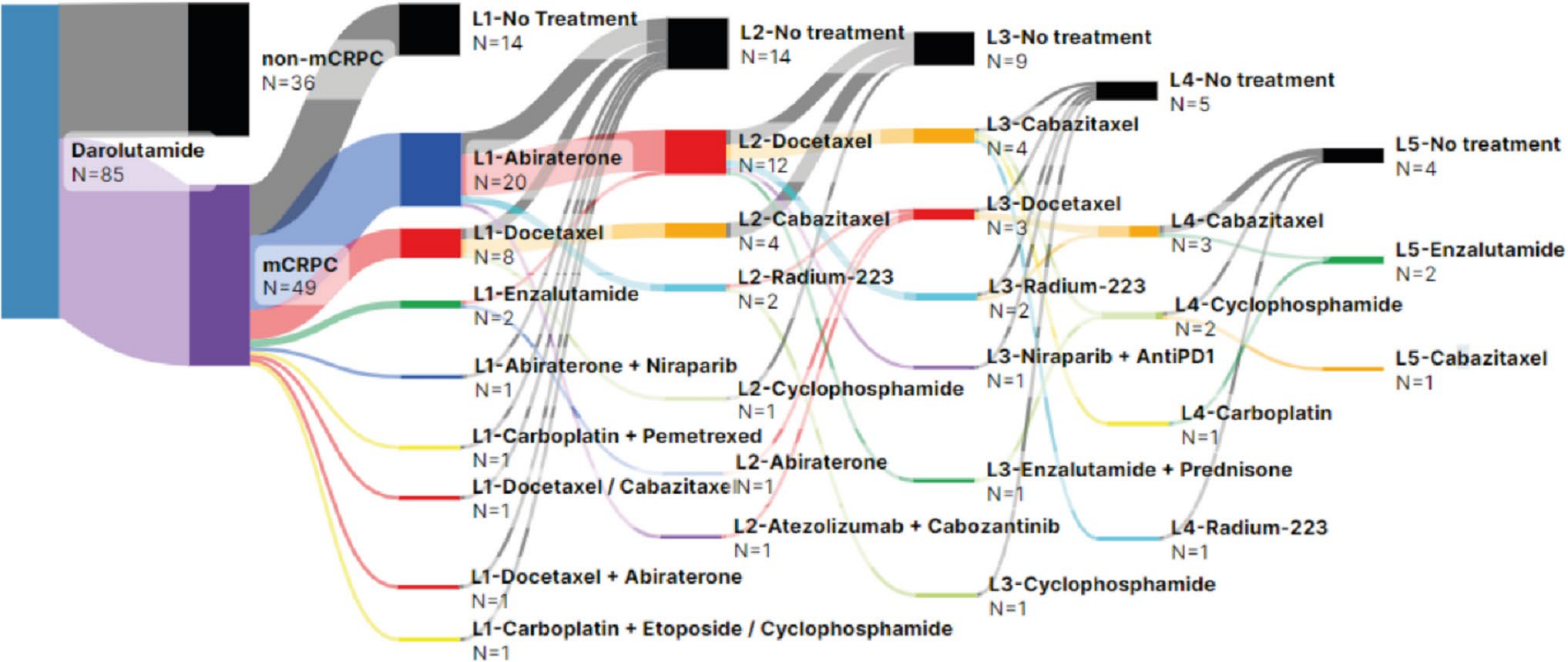
Sankey diagram of the mCRPC treatment line sequence after treatment with darolutamide. *L* line, *mCRPC* metastatic castration-resistant prostate cancer (nmCRPC)

The most frequent treatment sequence (first-line/second-line) for mCRPC was abiraterone–docetaxel (*n* = 20/*n* = 11) and docetaxel–cabazitaxel (*n* = 8/*n* = 4). The entire treatment line sequence is shown in Fig. 1.

### Duration of treatment after treatment with darolutamide and reasons for treatment discontinuation

The median (IQR) durations of abiraterone, enzalutamide, and docetaxel when administered first-line were 7.6 months (4.7, 12.7), 8.9 months (7.3, 10.6), and 4.8 months (3.8, 5.8), respectively. In addition, the corresponding median (IQR) durations for second-line docetaxel and cabazitaxel were 4.9 months (3.2, 6.3) and 2.9 months (1.4, 4.1), respectively. For third-line treatment, the durations were 5.1 months (4.3, 7.7) for docetaxel and 4.8 months (3.4, 9.4) for cabazitaxel, respectively.

Treatment discontinuation was ≥ 90% for all treatment lines. The main reasons for treatment discontinuation were disease progression (66.7–82.9%), death (5.7–14.3%), and drug-related toxicity (4.8–33.3%) (Supplementary Table 2).

### Radiotherapy and/or osteoclast-targeted therapies after treatment with darolutamide

Among the patients with metastatic disease, 11 (31.4%) patients received palliative radiotherapy (external beam radiotherapy) after treatment with darolutamide. Similarly, 7 patients (20%) were treated with osteoclast-targeted therapy (zoledronate or denosumab) during the follow-up, with a median (IQR) number of cycles of 26 (10.0, 42.0), and a median (IQR) duration of 12.9 months (8.8, 17.3).

### Progression to mCRPC by the last anticancer treatment

**Fig. 2:**
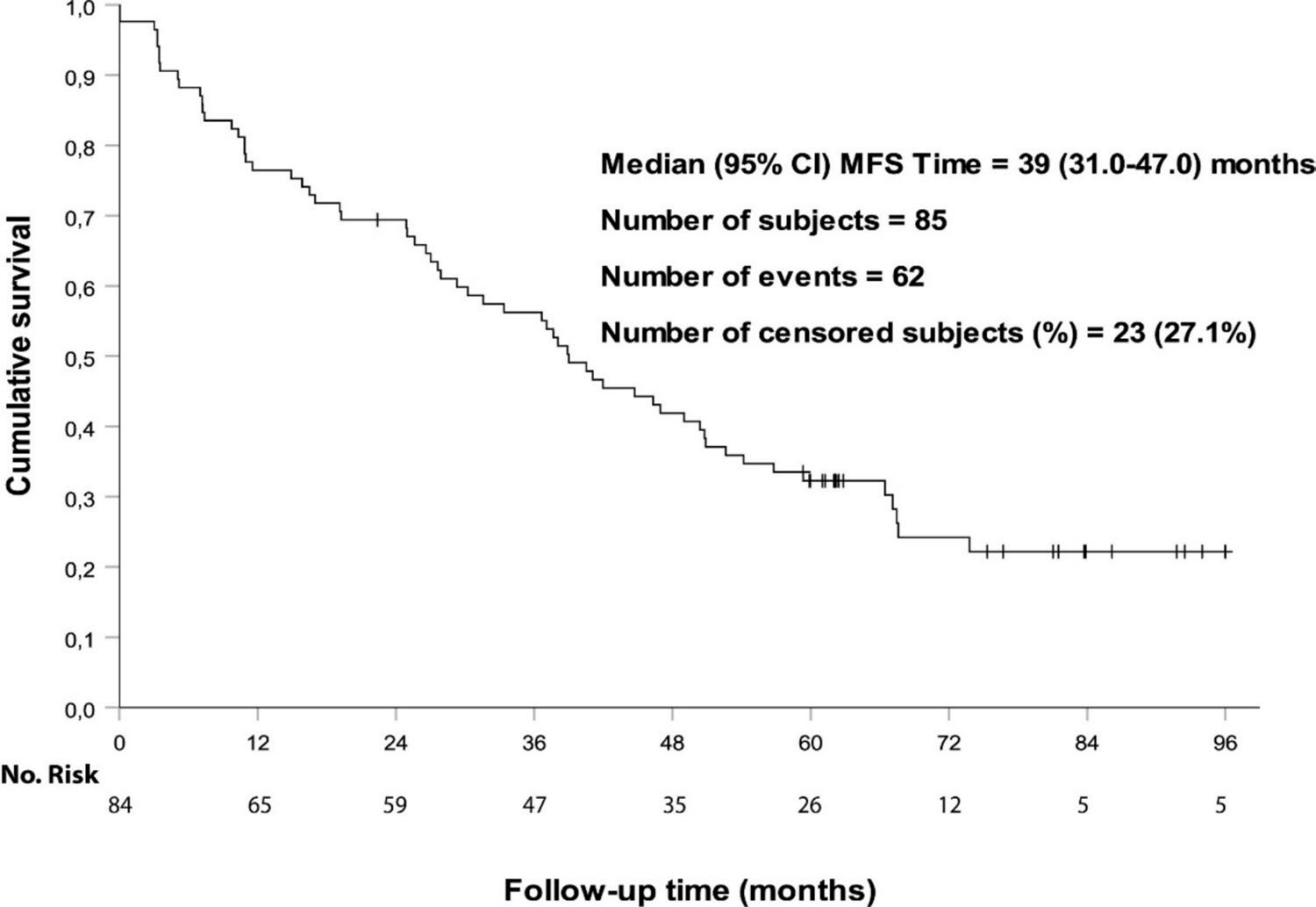
Metastasis-free survival. *MFS* metastasis-free survival

Forty-nine (57.6%) of the 85 included patients with ARAMIS/ROS progressed to mCRPC, with a median (IQR) number of metastatic locations of 2 (1.0, 4.0). The most common metastatic locations were bone in 34 patients (69.4%) and lymph nodes in 18 patients (36.7%) (Table 3). A Kaplan–Meier estimate revealed that the median (95% confidence interval (CI)) MFS time was 39 months (31.0–47.0) (Fig. 2).

**Table 3:** Metastatic locations and number of patients

Metastatic location	*n*	%^a^
Bone	34	69.4
Lymph nodes	18	36.7
Visceral	5	10.2
Lung	2	4.1
Pleural	1	2.0
Muscle	1	2.0
Chest	1	2.0
Brain	1	2.0
Abdominal	1	2.0

*n* absolute frequency

^a^Percentages calculated for all patients with metastatic locations (*N* = 49)

## Discussion

Our results show that almost 60% of the patients with nmCRPC treated with darolutamide progressed to mCRPC, with metastases located mainly in the bone. Among the patients with mCRPC, about 70% received at least one subsequent treatment, with the most common treatment being first-line abiraterone and the most common sequence (first-line/second-line) abiraterone–docetaxel and docetaxel–cabazitaxel. Only 20% of patients received osteoclast-targeted therapy.

In our study, we observed that 58% of patients with nmCRPC progressed to metastatic disease despite being previously treated with darolutamide, a result that is consistent with that reported in a post hoc analysis of the SPARTAN trial with apalutamide. Among the 548 patients who completed the trial, 311 (57%) patients developed mCRPC and subsequently received treatment [20]. However, in our study, nearly 30% of patients did not receive any therapies despite progressing to metastatic disease. This finding may have been due to a constellation of patient-related factors, such as old age (median of 76 years) and the presence of frailty and/or comorbidities, which might have contributed to hospital-associated functional decline in patients with prolonged hospital stays. In addition, there were marked differences in patient access and reimbursement for cancer therapies between Autonomous Communities in Spain, which could have contributed to this finding. Furthermore, among the 70% of patients who received treatment for mCRPC, the most commonly used first-line treatments in approximately two-thirds of patients, mostly consisted of new hormonal agents abiraterone + prednisone + ADT. This finding is not consistent with clinical practice guidelines [2, 6]. New hormonal agents, such as abiraterone and enzalutamide, and chemotherapy with docetaxel are considered first-line treatments for mCRPC [2, 6]; however, the use of a new hormonal treatment after another hormonal treatment, despite being the current clinical practice at the time the present study was conducted, is not consistent with clinical recommendations [2, 6]. Notably, our results are not consistent with those reported in other follow-up studies of patients treated with new hormonal therapies in the context of a clinical trial. Thus, in the PROSPER study, patients with nmCRPC received enzalutamide, followed most commonly by docetaxel, which was administered to 60% of patients [21]. Similarly, in the entire population of the ARAMIS trial, the most common treatment after having progressed to darolutamide was docetaxel in 58% of the patients [14]; in contrast, in our study, docetaxel was administered to 28% of the patients in the first-line after darolutamide. However, similar treatment patterns to our study have been reported by a large real-world US retrospective prostate cancer study using the nationwide Flatiron Health database in patients diagnosed with mCRPC [22]. ARPIs and chemotherapy were the first-line therapy in 75% and 16%, respectively, even though 71% of patients were ARPI-naïve before mCRPC diagnosis [22].

With respect to treatment sequence, the most common first- to second-line sequence in our study was abiraterone/docetaxel, which is in accordance with guidelines [2, 6] and has also been reported in real-world settings in Spain and other countries [23, 24]. Thus, in a large real-world study, the most common frequent sequence of first- and second-line treatment was NHA followed by chemotherapy, which was received by 35% of patients in the entire cohort (*n* = 469) and 30% of the patients in the Spanish subcohort (*n* = 96). Within that strategy, abiraterone–docetaxel was the most common sequence in the entire cohort (16%) and in the Spanish subcohort (17%) [23]. Similarly, most patients who were treated with docetaxel as first-line therapy were switched to cabazitaxel as a second-line therapy, a valuable option similarly endorsed by international guidelines [2, 3, 6] and reported in real-world settings [23].

In the PARASEC study, a very small percentage of patients were treated with PARP inhibitors (one patient with a combination of niraparib and abiraterone as first-line treatment), although this pharmacologic group has been shown to be effective in improving survival in patients with mCRPC harboring BRCA 1/2 mutations and other mutations that induce homologous recombination deficiency [25]. This finding may be related to either the access restrictions imposed by the time the patients included in the PARASEC study were treated, since the use of PARP inhibitors for mCRPC was not reimbursed by the Spanish national health care system, or due to the relatively low frequency of these mutations in patients with prostate cancer. In addition, next-generation sequencing of DNA was not available at all the participating sites. Similarly, in line with previous findings, very few patients were treated with radium-223 in the second, third, and fourth lines of treatment, despite its evidence-based benefit in prolonging the time to the first skeletal event [26].

Bone metastases are associated with highly debilitating symptoms, impaired quality of life, and poor prognosis [27]. Notably, although 70% of patients in the PARASEC study with mCRPC presented with bone metastasis, only 20% of the patients with mCRPC received osteoclast-targeted therapies, a finding that is not consistent with the guidelines’ recommendations [2] and reflects the clinical practice at the time the study was carried out.

All these real-world practice patterns, which are discordant with current guidelines, alert us of the need to comply with better standardized treatment algorithms and to optimize patient care across specialties through continuing medical education.

To the best of our knowledge, this is the first study to describe the treatment patterns in patients who progressed from nmCRPC to mCRPC following therapy with darolutamide in a real-world setting. However, this study has several limitations. We used a convenience sample with a relatively small size, which led to imprecision in our results, such as the MFS results. Due to its retrospective design, some data were not always available in the patients’ medical records. In addition, some selection bias cannot be ruled out, although it was minimized by identifying all the patients enrolled in the Spanish sites from the ARAMIS and ROS studies and by allowing all of them to participate in the PARASEC study. Furthermore, we included patients from both the ARAMIS and ROS studies, although the baseline characteristics of our study patients corresponded only to the date of inclusion in the ARAMIS trial; therefore, our study results can only be generalized to a population similar to that of the ARAMIS trial. We must also acknowledge the lack of genomic testing at the study sites, which precludes the guided use of novel efficacious drugs in specific subpopulations, such as PARP inhibitors in patients with mCRPC harboring BRCA 1/2 mutations. Likewise, we did not perform PSMA PET scans that would have allowed us to detect patients with PSMA-positive mCRPC who were susceptible to treatment with Lu-177-PSMA-617[6]. Both types of drugs, PARP inhibitors and Lu-11-PSMA-617 were not available in Spain at the time the study was conducted. Finally, as aforementioned, we cannot dismiss that Autonomous Community-related access restrictions may have biased treatment selection criteria among the participating centers in the PARASEC study.

In conclusion, our study shows that in Spain, one-third of patients treated with darolutamide who progress to metastatic castration-resistant prostate cancer do not receive treatment, and among those receiving treatment, a large proportion receive abiraterone, which appears not to be in line with the current European guidelines, although subsequent treatment sequencing seems in accordance with those guidelines. The use of osteoclast-targeted therapy is less common than expected considering the frequency of bone metastases. Overall, the variability in the duration of treatments and sequences suggests a lack of consensus in Spanish clinical practice for the management of patients with mCRPC in this setting and suggests that there is a need for more standardized strategies and unification of the criteria used in multidisciplinary committees to make collective and consensual decisions in accordance with the recommendations of international clinical practice.

## Supplementary Information

Below is the link to the electronic supplementary material.

Supplementary file1 (DOCX 96 KB)

## Data Availability

Data are available from the corresponding authors upon reasonable request.
