# Supplementary material for: Treatment patterns in patients with castration-resistant prostate cancer who received darolutamide in the ARAMIS trial in Spain: PARASEC study
Source: Clin Transl Oncol. 2026 Feb 10;28(8):3287–95. doi: 10.1007/s12094-026-04233-8 (PMC13401537; doi:10.1007/s12094-026-04233-8)
Supplement: Supplementary file 1 — Supplementary file1 (DOCX 96 KB) [file 12094_2026_4233_MOESM1_ESM.docx]

**Treatment patterns in patients with castration-resistant prostate cancer** **who received darolutamide in the ARAMIS trial in Spain: PARASEC study**

Javier Puente^a^, Rubén Campanario^b^, David Marmolejo^c^, Juan Andrés Cantero-Mellado^d^, Álvaro Gómez-Ferrer^e^, Alfredo Rodríguez Antolín^f^, María J. Ribal^g^, Natalia Picola Brau^h^, María José Ledo^i^, Carlos Hernandez^j^, Carlos Llorente^k^, Carmen González-Enguita^l^, Álvaro Bisono Castillo^m^, Joan Benejam^n^, Jesús Gil Guijarro^o^, Jose Garcia-Sanchez^p^, Joan Folqué^q^, Javier Casas-Nebra^r^

^a^Department of Medical Oncology, Hospital Clínico San Carlos, Madrid, Spain

^b^Department of Urology, Hospital Universitario Virgen del Rocío, Sevilla, Spain

^c^Department of Oncology, Hospital Universitari Vall d'Hebron, Barcelona, Spain

^d^Department of Urology, Hospital Clínico Universitario Virgen de la Victoria, Málaga, Spain

^e^Department of Urology, Instituto Valenciano de Oncología, Valencia, Spain

^f^Department of Urology, Hospital Universitario 12 de Octubre, Madrid, Spain

^g^Uro-oncology Unit, Hospital Clínic de Barcelona, Barcelona, Spain

^h^Department of Urology, Bellvitge University Hospital, Barcelona, Spain

^i^Department of Urology, Hospital Universitario Puerta del Mar, Cádiz, Spain

^j^Department of Urology, Gregorio Marañón University General Hospital, Madrid, Spain

^k^Department of Urology, Hospital Universitario Fundacion Alcorcon, Alcorcón, Spain

^l^Department of Urology, Hospital Universitario Fundación Jiménez Díaz, UAM, Madrid, Spain

^m^Hospital de Especialidades de Jerez De La Frontera

^n^Department of Urology, Hospital de Manacor, Mallorca, Spain

^o^Department of Urology, Hospital General Universitario de Elche

^p^Department of Oncology, Hospital Arnau de Vilanova, Valencia, Spain

^q^Bayer Hispania, S.L., Sant Joan Despí (Barcelona), Spain

^r^Department of Urology, Hospital Universitario Lucus Augusti, Lugo, Spain

**Corresponding author**

Javier Puente, MD, PhD

E-mail: javierpuente.hcsc@gmail.com

Department of Medical Oncology

Hospital Clínico San Carlos

C/ Profesor Martin Lagos s/n

28040-Madrid, Spain

Phone: +34913303000

**Supplementary information**

**Supplementary Table 1. Participating sites and patients enrolled by site**

| **Site** | **Number of patients** | **Percentage (%)** |
| --- | --- | --- |
| **Hospital Universitari Vall d'hebron (Barcelona)** | 8 | 9.4 |
| **Hospital Virgen de la Victoria (Málaga)** | 7 | 8.2 |
| **Hospital Lucus Augusti (Lugo)** | 7 | 8.2 |
| **Fundación Instituto Valenciano de Oncología** | 7 | 8.2 |
| **Hospital Universitario 12 de Octubre (Madrid)** | 6 | 7.1 |
| **Hospital Clínico San Carlos (Madrid)** | 6 | 7.1 |
| **Hospital Clínic de Barcelona (Barcelona)** | 6 | 7.1 |
| **HospitaL Universitari de Bellvitge (Barcelona)** | 5 | 5.9 |
| **Hospital Puerta del Mar (Cádiz)** | 4 | 4.7 |
| **Hospital General Universitario Gregorio Marañón (Madrid)** | 4 | 4.7 |
| **Hospital Universitario Fundación Jiménez Díaz (Madrid)** | 3 | 3.5 |
| **Hospital Universitario Fundación Alcorcón (Madrid)** | 3 | 3.5 |
| **Hospital de Especialidades de Jerez de la Frontera (Cádiz)** | 3 | 3.5 |
| **Hospital Manacor (Islas Baleares)** | 2 | 2.4 |
| **Hospital General Universitario de Elche (Alicante)** | 2 | 2.4 |
| **Hospital Arnau de Vilanova (Lérida)** | 2 | 2.4 |
| **Total** | 85 | 100 |

**Supplementary Table 2. Reasons for treatment discontinuation by treatment line**

| **Line number** | **Scheme Line** | **n** | **%** |
| --- | --- | --- | --- |
| **1** | Progression disease | 29 | 82.9 |
|  | Exitus | 2 | 5.7 |
|  | Toxicity | 2 | 5.7 |
|  | Patient starts palliative care | 1 | 2.9 |
|  | Ongoing treatment | 1 | 2.9 |
|  | **Total** | **35** | **100.0** |
| **2** | Progression disease | 14 | 66.7 |
|  | Exitus | 3 | 14.3 |
|  | Toxicity | 1 | 4.8 |
|  | Unknown | 1 | 4.8 |
|  | Ongoing Treatment | 2 | 9.5 |
|  | **Total** | **21** | **100.0** |
| **3** | Progression disease | 8 | 66.7 |
|  | Treatment finished | 1 | 8.3 |
|  | Investigator decision | 1 | 8.3 |
|  | Atypical pneumonia for cytomegalovirus | 1 | 8.3 |
|  | Ongoing Treatment | 1 | 8.3 |
|  | **Total** | **12** | **100.0** |
| **4** | Progression disease | 5 | 71.4 |
|  | Exitus | 1 | 14.3 |
|  | Not related adverse event | 1 | 14.3 |
|  | **Total** | **1** | **100.0** |
| **5** | Progression disease | 2 | 66.7 |
|  | Toxicity | 1 | 33.3 |
|  | **Total** | **3** | **100.0** |

Started treatment with darolutamide

| FPFV | Treatment after darolutamine | LPLV |
| --- | --- | --- |

| 2014 | 2015 | 2016 | 2017 | 2018 | 2019 | 2020 | 2021 | 2022 | 2023 | 2024 | 2025 |
| --- | --- | --- | --- | --- | --- | --- | --- | --- | --- | --- | --- |

Study period

FPFV: First patient first visit

LPLV: Last patient last visit

Study Period: May 2023 - Jan 2025

Start of data collection: Nov-23

End of data collection: may- 24

| May-23 | Jun-23 | Jul-23 | Aug-23 | Sep-23 | Oct-23 | Nov-23 | Dec-23 | Jan-24 | Feb-24 | Mar-24 | Apr-24 | May-24 | Jun-24 | Jul-24 | Aug-24 | Sep-24 | Oct-24 | Nov-24 | Dec-24 | Jan-25 |
| --- | --- | --- | --- | --- | --- | --- | --- | --- | --- | --- | --- | --- | --- | --- | --- | --- | --- | --- | --- | --- |

**Supplementary Figure 1. Study scheme**

**Supplementary Figure 2. Patient selection flow chart**
